# Reduzindo as Disparidades Sexuais nos Cuidados e Resultados do IAMCST: Virando a Maré para as Mulheres

**DOI:** 10.36660/abc.20220884

**Published:** 2023-01-31

**Authors:** F. Aaysha Cader, Cristina Gavina

**Affiliations:** 1 Departamento de Cardiologia Ibrahim Cardiac Hospital & Research Institute Dhaka Bangladesh Departamento de Cardiologia - Ibrahim Cardiac Hospital & Research Institute, Dhaka – Bangladesh; 2 Departamento de Cardiologia e Medicina Hospital Pedro Hispano Matosinhos Portugal Departamento de Cardiologia e Medicina - Hospital Pedro Hispano, ULS Matosinhos – Portugal; 3 Universidade do Porto Porto Portugal Universidade do Porto, Porto - Portugal

**Keywords:** Infarto do Miocárdio com supradesnivelamento do segmento ST, Disparidades Sexuais, Mulheres, Síndromes Coronarianas Agudas

Apesar das melhorias no tratamento das síndromes coronarianas agudas (SCA), globalmente, as disparidades entre os sexos permanecem.^1^ As mulheres com infarto do miocárdio com supradesnivelamento do segmento ST (IAMCST) apresentam-se mais tarde, recebem cuidados abaixo do ideal e têm resultados piores do que os homens.^1 - 3^

Em uma análise retrospectiva de centro único de 809 pacientes com IAMCST submetidos a ICP primária em Braga, Portugal, Oliveira et al. relataram disparidades sexuais na apresentação, tratamento e resultados.^4^ Embora a incidência de doença isquémica cardíaca e morte coronária seja menor em Portugal do que em outros países europeus,^5^ a morte cardiovascular continua a ser a principal causa de mortalidade.

De modo semelhante às tendências globais, neste estudo, as mulheres eram mais velhas, com maior prevalência de comorbidades, incluindo diabetes, hipertensão, doença renal crônica e acidente vascular cerebral isquêmico agudo, e um escore de risco GRACE mais alto.^4^ As mulheres apresentaram mais frequentemente “sintomas atípicos”, pior classe de Killip, choque cardiogênico e necessidade de suporte hemodinâmico. Elas também experimentaram maior atraso na reperfusão e eram menos propensas a receber terapia médica recomendada pelas diretrizes internacionais.^4^ As mulheres tiveram maior mortalidade intra-hospitalar, de curto e longo prazo em 1 ano, em comparação aos homens, embora o sexo feminino não tenha sido um fator prognóstico independente para mortalidade. Os piores resultados foram observados com o aumento da idade, embora uma análise específica estratificada por sexo e idade não tenha sido apresentada.^4^

Estas disparidades de sexo refletem os registos globais e o grande Registo Português de Síndromes Coronárias Agudas (ProACS), incluindo 49.113 doentes com SCA, 14.177 dos quais eram mulheres.^6^ Além de confirmar as diferenças já descritas, este registro também relatou uma alta incidência de sangramento maior e fibrilação atrial.

A revascularização imediata é de suma importância para melhores resultados em IAMCST. Neste estudo, atrasos significativamente maiores foram observados em todos os parâmetros para as mulheres depois que elas se apresentaram para atendimento médico, incluindo primeiro contato médico (PMC) para eletrocardiograma, início dos sintomas para balão e PMC para balão.^4^ Notavelmente, não foram observadas diferenças no tempo desde o início dos sintomas até a PMC, apesar do relato de sintomas mais “atípicos” pelas mulheres. Isso indica potencialmente vieses implícitos nos cuidados de saúde e um sub-reconhecimento dos sintomas, provavelmente resultando em tratamento menos agressivo das mulheres.^7^

De fato, a literatura contemporânea demonstra que os sintomas nas mulheres são frequentemente mais semelhantes aos dos homens,^8 , 9^ com aproximadamente 90% das mulheres e homens com infarto do miocárdio (IM) relatando dor torácica como sintoma inicial.^10^ No presente estudo, as mulheres relataram mais sintomas “atípicos” de dor epigástrica, náusea e dispneia.^4^ O reconhecimento das diferenças entre os sexos nos sintomas da SCA é fundamental, pois as mulheres geralmente relatam três ou mais sintomas acompanhantes junto com desconforto torácico.^10^ Terminologia como “dor torácica atípica” se presta a cuidados menos emergentes em mulheres e, portanto, não é mais recomendada como uma palavra para descrever a dor torácica pelas Diretrizes de Dor Torácica de 2021 da American Heart Association e do American College of Cardiology.^11^

As mulheres submetidas à ICP primária frequentemente apresentam sangramento maior, com estratégias de reperfusão menos frequentes envolvendo tromboaspiração e acesso transradial,^12^ o que é conhecido por reduzir o sangramento e melhorar a mortalidade na SCA. Este aspecto, bem como variáveis relacionadas com o procedimento, como a composição da placa na imagem intravascular, ou a proporção de infarto do miocárdio com artérias coronárias não obstrutivas (MINOCA) ou dissecção espontânea da artéria coronária (SCAD),^13 , 14^ ambas as mais prevalentes de IAMCST em mulheres, não foram relatadas nesta análise.

O sucesso da ICP primária depende do grau de carga de trombo no vaso ocluído e sua embolização distal a jusante, levando a fluxo lento ou não-refluxo.^15^ Uma metanálise recente de três estudos randomizados descobriu que, entre os pacientes com IAMCST com alta carga de trombos (HTB), as mulheres tinham maior risco de trombose de stent e mortalidade cardiovascular e por todas as causas do que os homens.^15^ Embora o HTB não tenha sido capturado neste estudo, as mulheres eram significativamente mais propensas a ter um grau de fluxo pré-PCI TIMI de 0 e aumento do fluxo lento/não-refluxo.^4^ Apesar disso, o uso de heparina não fracionada foi significativamente menor em mulheres do que em homens (85,4% vs. 90,0%).

Embora o sexo não tenha sido considerado um preditor independente de mortalidade após o ajuste para covariáveis, as disparidades no atendimento em geral levam a um forte apelo à ação no IAMCST e, de fato, no cuidado de mulheres com SCA.

Como podemos virar a maré? Globalmente, existem vieses implícitos nos cuidados de saúde, resultando em subdiagnóstico e atraso no tratamento de mulheres com IAMCST. Mensagens apropriadas de saúde pública para garantir a apresentação oportuna em hospitais^2^ e educação e treinamento de mitigação de viés do pessoal de saúde são de suma importância, assim como atenção apropriada para reconhecer sintomas acompanhantes de dor torácica.^1^

Abordagens baseadas em protocolo e baseadas em sistemas para tratamento de IAMCST demonstraram reduzir as disparidades entre sexos no tratamento de IAMCST e melhorar os resultados em mulheres^2^ e devem ser incorporadas à prática. A representação feminina em ensaios clínicos deve ser reforçada. Existem disparidades regionais nas percepções e práticas; portanto, é necessária uma pesquisa específica da região e do país, alavancando registros em larga escala para identificar lacunas na gestão e preditores específicos do sexo dos resultados do IAMCST, para melhor definir abordagens direcionadas ao atendimento, que são mais adequadas ao sistema de saúde de cada país.
