# Supplementary material for: Reduzindo as Disparidades Sexuais nos Cuidados e Resultados do IAMCST: Virando a Maré para as Mulheres
Source: Arq Bras Cardiol. 2023 Jan 31;120(1):e20220884. [Article in Portuguese] doi: 10.36660/abc.20220884 (PMC10389104; doi:10.36660/abc.20220884)
Supplement: Translation

# Translation: Reducing Sex Disparities in STEMI Care and Outcomes: Turning the Tide for Women

**Authors:** F. Aaysha Cader, Cristina Gavina

**Affiliations:** 1 Bangladesh Departamento de Cardiologia - Ibrahim Cardiac Hospital & Research Institute, Dhaka – Bangladesh; 2 Portugal Departamento de Cardiologia e Medicina - Hospital Pedro Hispano, ULS Matosinhos – Portugal; 3 Portugal Universidade do Porto, Porto – Portugal

**Keywords:** ST Elevation Myocardial Infarction, Sex Disparities, Women, Acute Coronary Syndromes

Despite improvements in acute coronary syndromes (ACS) care, globally, sex disparities remain.^1^Women with ST-segment elevation myocardial infarction (STEMI) present later, receive suboptimal care, and have worse outcomes than men.^1 - 3^

In a single-center retrospective analysis of 809 STEMI patients undergoing primary PCI in Braga, Portugal, Oliveira et al. reported sex disparities in presentation, management, and outcomes.^4^Although ischemic heart disease and coronary death incidence are lower in Portugal than in other European countries,^5^cardiovascular death remains the main cause of mortality.

Similar to global trends, in this study, women were older, with a greater prevalence of co-morbidities, including diabetes, hypertension, chronic kidney disease, and acute ischemic stroke, and a higher GRACE risk score.^4^Women presented more frequently with “atypical symptoms,” worse Killip class, cardiogenic shock, and required hemodynamic support. They also experienced delayed reperfusion and were less likely to receive guideline-directed medical therapy.^4^Women had greater in-hospital, short and long-term mortality at 1 year, compared to men, although the female sex was not an independent prognostic factor for mortality. Worse outcomes were seen with increasing age, although a sex-specific analysis of outcomes by age was not reported.^4^

These sex disparities reflect global registries and the large Portuguese Registry of Acute Coronary Syndromes (ProACS), including 49,113 ACS patients, 14,177 of whom were women.^6^In addition to the outcomes above, this registry also reported a higher incidence of major bleeding and atrial fibrillation.

Prompt revascularization is of paramount importance for improved outcomes in STEMI. In this study, significantly greater delays were seen in all parameters for women after they had presented for medical care, including first medical contact (FMC)-to-electrocardiogram, symptom onset-to-balloon, and FMC-to-balloon.^4^Notably, no differences were seen in time from symptom onset to FMC, despite women reporting more “atypical” symptoms. This potentially indicates implicit biases in healthcare, and an under-recognition of symptoms, likely resulting in less aggressive treatment of women.^7^

Indeed, contemporary literature demonstrates that symptoms in women are often more similar to men,^8 , 9^with approximately 90% of women and men with a myocardial infarction (MI) reporting chest pain as the presenting symptom.^10^In the present study, women reported more “atypical” symptoms of epigastric pain, nausea, and dyspnea.^4^Recognition of sex differences in symptoms of ACS is paramount, as women often report three or more accompanying symptoms along with chest discomfort.^10^Terminology such as “atypical chest pain” lends itself to less emergent care in women and is thus no longer recommended as a word to describe chest pain by the 2021 Chest Pain Guidelines from the American Heart Association and the American College of Cardiology.^11^

Women undergoing primary PCI frequently experience more major bleeding, with reperfusion strategies less frequently involving thrombo-aspiration and transradial access,^12^which is known to reduce bleeding and improve mortality in ACS. This aspect, as well as additional procedural variables such as plaque composition on intravascular imaging, proportions of myocardial infarction with non-obstructive coronary arteries (MINOCA) or spontaneous coronary artery dissection (SCAD),^13 , 14^both of which are more prevalent STEMI presentations in women, were not reported in this analysis.

The success of primary PCI hinges on the degree of thrombus burden in the occluded vessel and its downstream distal embolization, leading to slow flow or no-reflow.^15^A recent meta-analysis of three randomized trials found that among STEMI patients with the high-thrombus burden (HTB), women had more risk for stent thrombosis and cardiovascular and all-cause mortality than men.^15^Although HTB was not captured in this study, women were significantly more likely to have a pre-PCI TIMI flow grade of 0 and increased slow flow/no-reflow.^4^Despite this, unfractionated heparin use was significantly less prevalent in women than in men (85.4% vs. 90.0%).

Although sex was not found to be an independent predictor of mortality after adjustment for covariates, the disparities in care across the board prompt a louder call for action in STEMI and, indeed, ACS care of women.

How do we turn the tide? Globally, implicit biases in healthcare exist, resulting in underdiagnosis and delayed treatment of women with STEMI. Appropriate public health messaging to ensure timely presentation at hospitals^2^and education and bias mitigation training of healthcare personnel are of paramount importance, as is appropriate attention to recognizing accompanying symptoms of chest pain.^1^

Protocol-driven, systems-based approaches to STEMI care have demonstrated reduced sex disparities in STEMI care and improved outcomes in women^2^and should be incorporated into practice. Female participant representation in clinical trials must be enhanced. Regional disparities in perceptions and practice exist; therefore, region and country-specific research is needed, leveraging large-scale registries to identify gaps in management and sex-specific predictors of STEMI outcomes to define better-targeted approaches to care best suited to each country’s healthcare system.
